# Feasibility and comparability of different platelet function tests in acute stroke with or without prior antiplatelet therapy

**DOI:** 10.3389/fneur.2024.1361751

**Published:** 2024-02-12

**Authors:** Jan Hendrik Schaefer, Franziska Lieschke, Hans Urban, Ferdinand O. Bohmann, Florian Gatzke, Wolfgang Miesbach

**Affiliations:** ^1^Department of Neurology, University Hospital Frankfurt, Goethe University, Frankfurt am Main, Germany; ^2^Department of Internal Medicine II, Haemostaseology and Haemophilia Centre, University Hospital Frankfurt, Goethe University, Frankfurt am Main, Germany

**Keywords:** platelets, thrombocytes, FACS, aggregometry, platelet functioning analyzer, acute stroke, ischemic stroke, hemorrhagic stroke

## Abstract

**Background:**

The clinical course of ischemic and hemorrhagic strokes can be influenced by the coagulation status of individual patients. The prior use of antiplatelet therapy (APT) such as acetylsalicylic acid (ASA) or P2Y12-antagonists has been inconsistently described as possibly increasing the risk of hemorrhagic transformation or expansion. Since clinical studies describing prior use of antiplatelet medication are overwhelmingly lacking specific functional tests, we aimed to implement testing in routine stroke care.

**Methods:**

We used fluorescence-activated cell sorting (FACS) with antibodies against CD61 for thrombocyte identification and CD62p or platelet activation complex-1 (PAC-1) to determine platelet activation. Aggregometry and automated platelet functioning analyzer (PFA-200) were employed to test thrombocyte reactivity. FACS and aggregometry samples were stimulated *in vitro* with arachidonic acid (AA) and adenosine diphosphate (ADP) to measure increase in CD62p-/PAC-1-expression or aggregation, respectively.

**Results:**

Between February and July 2023, 20 blood samples (*n* = 11 ischemic strokes; *n* = 7 hemorrhagic strokes; *n* = 2 controls) were acquired and analyzed within 24 h of symptom onset. *N* = 11 patients had taken ASA, *n* = 8 patients no APT and *n* = 1 ASA+clopidogrel. ASA intake compared to no APT was associated with lower CD62p expression after stimulation with AA on FACS analysis (median 15.8% [interquartile range {IQR} 12.6–37.2%] vs. 40.1% [IQR 20.3–56.3%]; *p* = 0.020), lower platelet aggregation (9.0% [IQR 7.0–12.0%] vs. 88.5% [IQR 11.8–92.0%]; *p* = 0.015) and longer time to plug formation with PFA-200 (248.0 s [IQR 157.0–297] vs. 121.5 s [IQR 99.8–174.3]; *p* = 0.027). Significant correlations were noted between AA-induced CD62p expression and aggregometry analysis (*n* = 18; ρ = 0.714; *p* < 0.001) as well as a negative correlation between CD62p increase and PFA clot formation time (*n* = 18; ρ = −0.613; *p* = 0.007). Sensitivity for ASA intake was highest for PFA (81.8% for values ≥155.5 s). The combination of ASA + clopidogrel also affected ADP-induced CD62p and PAC-1 expression.

**Conclusion:**

In the clinical setting it is feasible to use differentiated platelet analytics to determine alterations caused by antiplatelet therapy. Among the tests under investigation, PFA-200 showed the highest sensitivity for the intake of ASA in stroke patients. FACS analysis on the other hand might be able to provide a more nuanced approach to altered platelet reactivity.

## Introduction

In synchrony with other vascular diseases, the use of antiplatelet therapy (APT) in the form of acetylsalicylic acid (ASA) or P2Y12 inhibitors such as clopidogrel and ticagrelor has become a mainstay in the prevention of ischemic stroke (1, 2). For minor ischemic stroke and high risk transient ischemic attacks (TIAs), the administration of dual antiplatelet therapy (DAPT) for 21–90 days has recently been established as a way to further decrease the risk of recurrent stroke, albeit with an increased risk of hemorrhage (3, 4). So far, the individual risk of hemorrhage is difficult to predict. Particularly, in cases of recurrent ischemic stroke or hemorrhagic stroke the literature on possibly deleterious effects of pre-treatment with single or dual antiplatelet medication yields heterogenous results.

In ischemic stroke, the use of thrombolysis for ischemic stroke patients on antiplatelet medication, especially in combination, has been suggested to increase the risk of symptomatic hemorrhage in some studies (5–7) whereas other authors have concluded that no significant difference in symptomatic hemorrhage can be observed, and more importantly, the outcome was independent of prior APT (8, 9).

Similarly, the outcome of hemorrhagic stroke in patients with prior APT has been described at variance with either increased mortality, but equal functional outcomes (10), no effects if not combined with vitamin K antagonists (11), or even increased hematoma expansion and worse outcomes (12). A common problem in these study designs is the reliability of patient history when it comes to the regular intake of APT as well as possible intraindividual effects on platelet reactivity. The latter might manifest as potential APT-non-responders, which have been identified in previous studies as at risk for neurological deterioration after stroke (13, 14).

Unlike thrombocyte counts, the platelet function is oftentimes not measured in standard care for stroke or large trials. Yet, there are several measuring methods, among which light-transmission aggregation (LTA) is frequently cited as the gold standard and can monitor APT in patients (15). In the past, automatized methods such as point-of-care Multiplate devices measuring platelet aggregometry via impedance changes have been studied in patients with acute brain injury, showing a significant proportion of platelet inhibition with and without known APT, which led to a targeted hemostatic therapy in the majority of patients (16). Another method utilizes an automated platelet-functioning-analyzer (PFA), which uses high shear forces to measure a closure time of a coated aperture but may show wide variations in APT response (16, 17). Progressively, the use of fluorescence-activated cell sorting has been researched in clinical and preclinical settings to determine the effects on APT (18, 19).

In this study, we aimed to evaluate the feasibility and comparability of different platelet functioning tests in patients with acute ischemic or hemorrhagic strokes presenting to a tertiary stroke center and correlate the results with prior intake of APT before stroke.

## Methods

The protocol was designed as a monocentric, prospective biomarker study, performed at the University Hospital Frankfurt, Goethe University. Inclusion criteria were (1) age ≥ 18 years, (2) diagnosis of an acute ischemic or hemorrhagic stroke, confirmed by brain imaging (computed tomography or magnetic resonance tomography), and hospital admission within 24 h of symptom onset. Groups were classified according to prior intake of antiplatelet therapy or presumed normal coagulation status. Healthy volunteers with or without prior antiplatelet therapy served as controls. Exclusion criteria were therapy with vitamin K antagonists or direct oral anticoagulants, relevant thrombocytopenia (< 100/nL) or administration of intravenous thrombolysis with recombinant tissue plasminogen activator prior to blood sampling as well as known coagulation disorders such as von Willebrand disease or hemophilia. After study inclusion, blood samples (15–20 mL) were drawn and immediately mixed with 0.106 mol/L sodium citrate by slowly inverting the tube to prevent coagulation. Within 90 min (min) samples were transported to the local specialized coagulation laboratory for platelet-functioning analyzer (PFA-200) and thrombocyte aggregometry, which was completed within 180 min of venipuncture. The PFA-200 examines closure times in seconds after aspiration of whole blood through disposable test cartridges, which are coated with epinephrine resulting in a plug formation inside a microscopic aperture (20). To measure platelet aggregation, light transmission aggregometry (LTA) was performed. The principle of the test involves measuring the photometric properties of platelet-rich plasma (PRP) after it has undergone platelet aggregation due to the action of certain activators. This process of *in vitro* platelet aggregation is a result of platelet activation triggered by different soluble activators. The activation process engages specific membrane receptors and triggers downstream signaling pathways, as well as amplification pathways. These pathways and their intensity vary depending on both the kind and quantity of the activator introduced. For this, arachidonic acid was added to platelet-rich plasma and thrombocyte aggregation is detected via changes in light transmission and measured as percentage (21).

For FACS measurement, blood samples were within 5–15 min of venipuncture centrifuged at 120 g without brake for 10 min. After spinning, 35 μL of platelet-rich-plasma (PRP) was transferred into tubes containing 15 μL phosphate-buffered saline (PBS), containing 0.32% sodium citrate. To initiate thrombocyte activation either 4 μl arachidonic acid (AA; concentration 15 mmol/L; Hyphen BioMed; item no. AG003K) or 4 μl adenosine diphosphate (ADP; concentration 20 μmol/L; Hyphen BioMed; item no. AG001K) were added to the diluted PRP and incubated at 37°C for 5 min. A subset of samples was also stimulated by 4 μl of thrombin (concentration 100 U/mL; Sigma Aldrich; item no. T6884-100UN) to test the maximum of activation. To inhibit fibrinogen polymerization, 10 mM Gly-Pro-Arg-Pro (GPRP; Sigma Aldrich; item no. G1895-25MG) was added before stimulation with thrombin. Afterwards, samples were stained using 10 μl of fluorescent anti-human antibodies against CD61 (fluorochrome PerCP) as a platelet-specific marker, CD62p (fluorochrome PE), also known as P-selectin, which is externalized upon platelet-activation, or platelet activation complex-1 (PAC-1) (fluorochrome FITC), which targets the active glycoprotein IIb/IIIa complex. Additionally, corresponding isotype controls (antibodies and isotype controls all by BD Biosciences) were used to distinguish possible binding to unspecific cells. To ensure specific PAC-1 binding, a subset of samples included 10 μl Arg-Gly-Asp-Ser (RGDS; Abcam; item no. 230365) solution, which competitively inhibits PAC-1 binding (22).

The samples were then incubated for 20 min at room temperature in the dark. Afterwards, samples were fixed by adding 650 μl cold fixative solution (650 μl PBS containing 0.1% paraformaldehyde, 0.1% glucose, and 0.2% bovine serum albumin to a final volume of 720 μl). After incubation for 60 min at 2°C−8°C, protected from light, all samples were analyzed with a FACS Canto II device (BD Biosciences) within 5 h of venipuncture.

Statistical analyses were performed with the Statistical Package for the Social Sciences (SPSS, version 27.0.1.0, Armonk, N.Y., USA) and GraphPad Prism (Version 10.1.0, Boston, MA, USA). The Kolmogorov-Smirnov-test was used to test for normal distribution. Categorical data were assessed for significance by Pearson-χ^2^-tests, continuous variables were analyzed using the Mann–Whitney–*U*-test for two independent samples. The results of the different platelet test methods were compared calculating Pearson correlation coefficients. A receiver-operator-characteristics (ROC) curve analysis was performed in order to obtain sensitivity values in regard to the presence of APT. All tests were two-sided and statistical significance was set to *p* < 0.05. FACS data were analyzed with Flowjo (version 10.9.0, BD Biosciences, Franklin Lakes, N.J., USA).

The study was conducted in accordance with the Declaration of Helsinki and approved by the local Ethics Committee of the Goethe University Frankfurt (2022-989). Written informed consent was provided by all patients before enrolment. The study was registered in the German Clinical Trials Register (ID DRKS00030574; https://www.drks.de).

## Results

### Study population

Between February and July 2023, 20 blood samples (*n* = 11 ischemic strokes; *n* = 7 hemorrhagic strokes; *n* = 2 controls) were acquired. *N* = 11 patients had taken ASA, *n* = 8 patients no APT and *n* = 1 ASA and clopidogrel. Mean age was 63.4 ± 15.6 years and 30% were female (Table 1). The median time from symptom onset to hospital admission was 08 h 23 min.

**Table 1:** Baseline characteristics of all patients, ischemic and hemorrhagic strokes as well as healthy controls.

	**All**	**Ischemic strokes**	**Hemorrhagic strokes**	**Healthy controls**
*N*	20	11	7	2
Age (years; mean ± SD)	63.4 ± 15.6	71.8 ± 10.2	58.4 ± 12.6	34.5 ± 0.7
Sex (female:male)	6:14	3:8	3:4	0:2
Median time from symptom onset (hours; IQR)	5.5 (1.7–12.0)	8.3 (1.7–12.0)	4.6 (2.3–10.3)	na
NIHSS at admission (median; IQR)	5.0 (1.0–19.0)	4.0 (1.0–8.0)	12.0 (5.0–17.0)	na
ASA pretreatment (*n*; %)	12 (60%)	7 (63.6%)	4 (57.1%)	1 (50%)
Clopidogrel pretreatment (*n*; %)	1 (5%)	1 (9.0%)	0 (0%)	0 (0%)
Arterial hypertension (*n*; %)	15 (75%)	10 (90.9%)	5 (71.4%)	0 (0%)
Diabetes (*n*; %)	5 (25%)	4 (36.3%)	1 (14.3%)	0 (0%)
Coronary heart disease (*n*; %)	5 (25%)	2 (18.2%)	3 (42.9%)	0 (0%)
mRS at discharge (median; IQR)	2 (1.0–4.0)	1 (0.5–2.5)	5 (3.0–5.0)	na

SD, standard deviation; NIHSS, National Institutes of Health Stroke Scale; ASA, acetylsalicylic acid; mRS, modified Rankin Scale; na, not applicable.

### Platelet activation measurements

**Figure 1:**
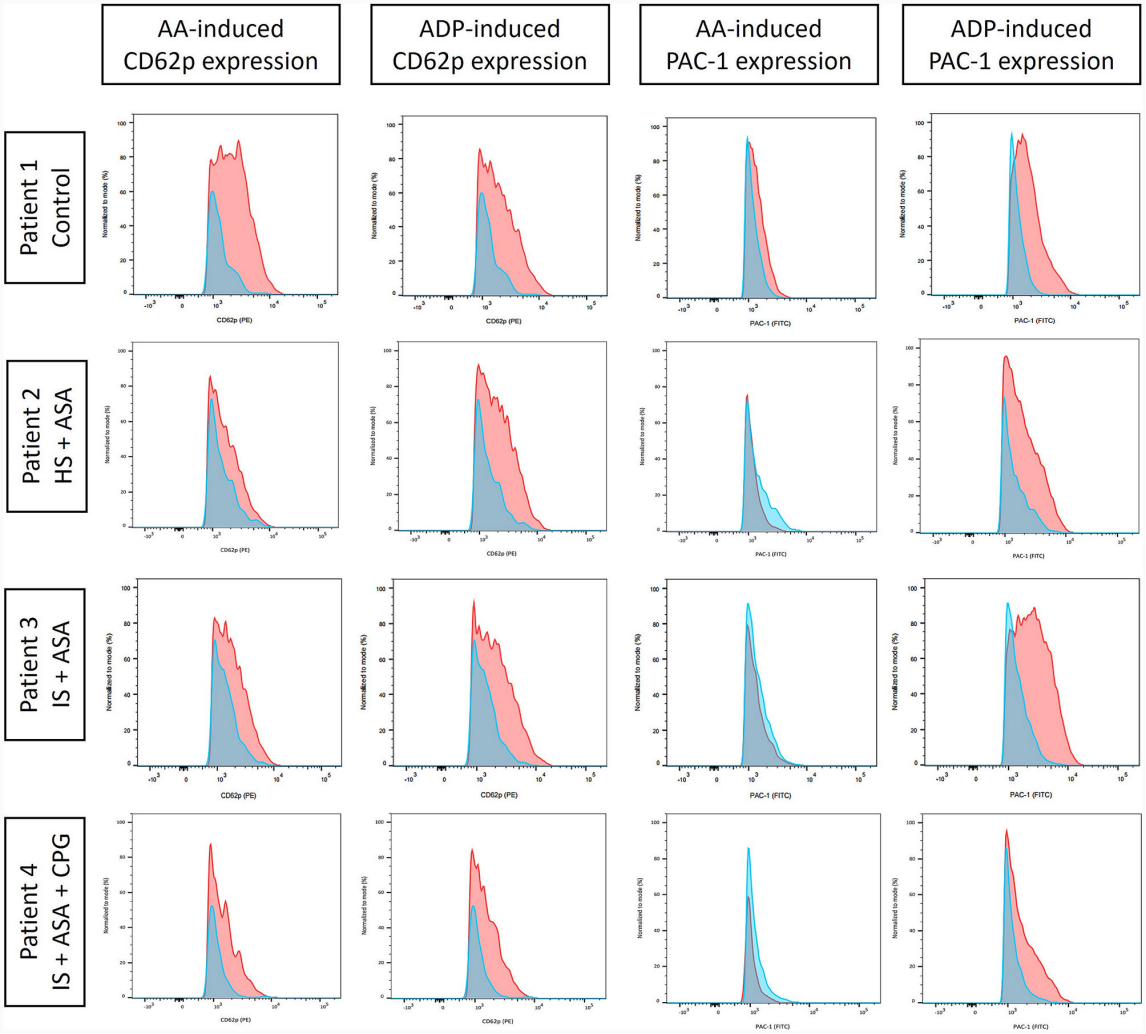
Fluorescence-activated cell sorting (FACS) analysis of CD62p and platelet activation complex-1 (PAC-1) expression as proportion of CD61-positive cells (all thrombocytes) signifying platelet activation in unstimulated samples (blue) and after stimulation (red) induced by arachidonic acid (AA) or adenosine diphosphate (ADP). The values were normalized to mode to facilitate comparability. Exemplarily, four patients are shown [control; hemorrhagic stroke (HS); ischemic stroke (IS)] with different antiplatelet therapy: none (control), acetylsalicylic acid (ASA), and clopidogrel (CPG). Differences in AA-induced platelet reactivity were noted for ASA intake, whereas CPG chiefly reduced ADP-induced platelet activation.

**Figure 2:**
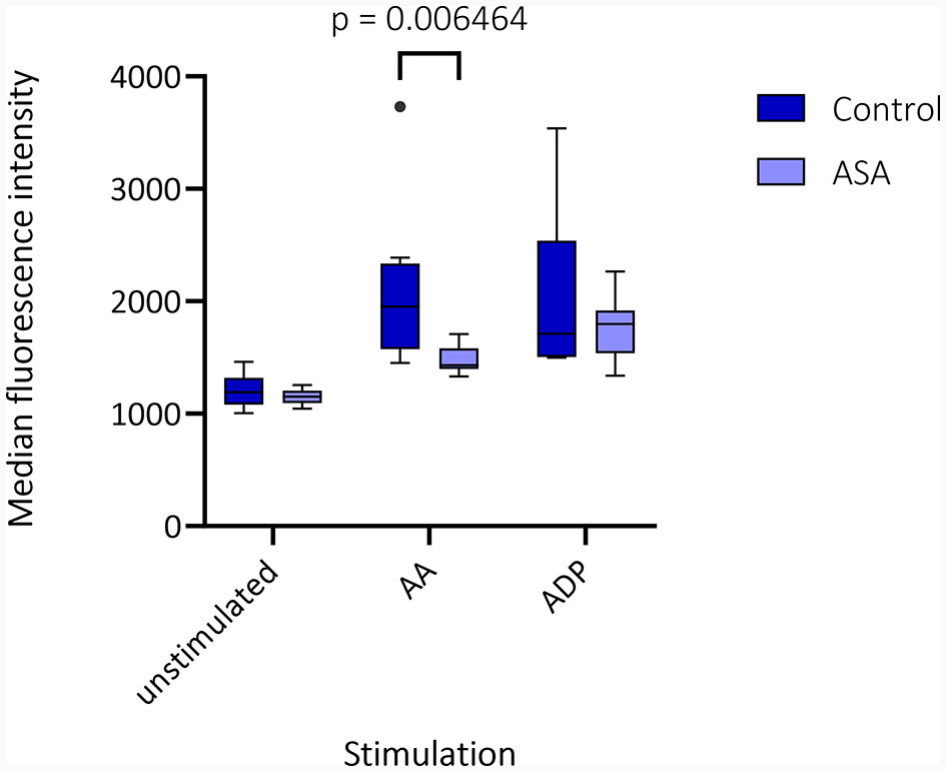
Median fluorescence intensity (MFI) in fluorescence-activated cell sorting (FACS) is depicted as box plots for CD62p-expression in unstimulated samples and after stimulation with arachidonic acid (AA) and adenosine diphosphate (ADP) for patients without (control) or with acetylsalicylic acid (ASA) pretreatment. Multiple Mann–Whitney–*U*-tests for group differences revealed significant higher values for CD62p-MFI in the absence of antiplatelet therapy compared to the ASA group (mean ± standard deviation 2103 ± 734 vs. 1477 ± 117; *p* = 0.006).

Median time from venipuncture to start of FACS measurements was 168 min (interquartile range [IQR] 159.5–206.0 min). Visual inspection of CD62p-expression with and without AA- and ADP-stimulation demonstrated differences in regard to medication with ASA and clopidogrel (Figure 1). Unstimulated, CD62p-expression was measured as median 2.21% (IQR 0.98–5.2%) without significant difference between hemorrhagic and ischemic strokes (median 1.26% [IQR 1.19–2.74] vs. 2.27 [IQR 0.8–5.41]; *p* = 0.68). Notably, ASA intake compared to no APT was associated with lower CD62p expression after stimulation with AA (15.8%; IQR 12.6–37.2% vs. 40.1%; IQR 20.3–56.3%; *p* = 0.020). Additionally, the median fluorescence intensity (MFI) was calculated for CD62p-expression. Mann–Whitney–*U*-test for group differences revealed significant higher values for CD62p-MFI in the absence of APT compared to the ASA group (mean ± standard deviation 2103 ± 734 vs. 1477 ± 117; *p* = 0.006; Figure 2). PAC-1 specificity of antibodies was confirmed with RGDS-inhibition (*n* = 7) leading to a complete suppression of PAC-1 expression despite ADP-stimulation. No significant differences for PAC-1 expression were observed between unstimulated and AA-stimulated samples (median 7.18%; IQR 3.86–12.80% vs. 5.87%; IQR 3.15–12.40%; *p* = 0.62). ADP-stimulation resulted in a significant increase of PAC-1 positive cells (median 47.30%; IQR 32.6–52.6%, *p* < 0.0001), but no difference between the ASA-group and patients without APT was detected in this setting (median 48.0%; IQR 32.55–55.43% vs. 44.85%; IQR 33.0–52.55; *p* = 0.79). The exploratory use of thrombin to stimulate platelet activation resulted in an almost complete expression of CD62p-positive cells (*n* = 4; median 85.45%, IQR 79.0–90.6%) and a majority of PAC-1-positive cells (median 70.55%; IQR 57.5–80.8%) as well as reaching 98% respectively in a subject with confirmed ASA-intake.

**Figure 3:**
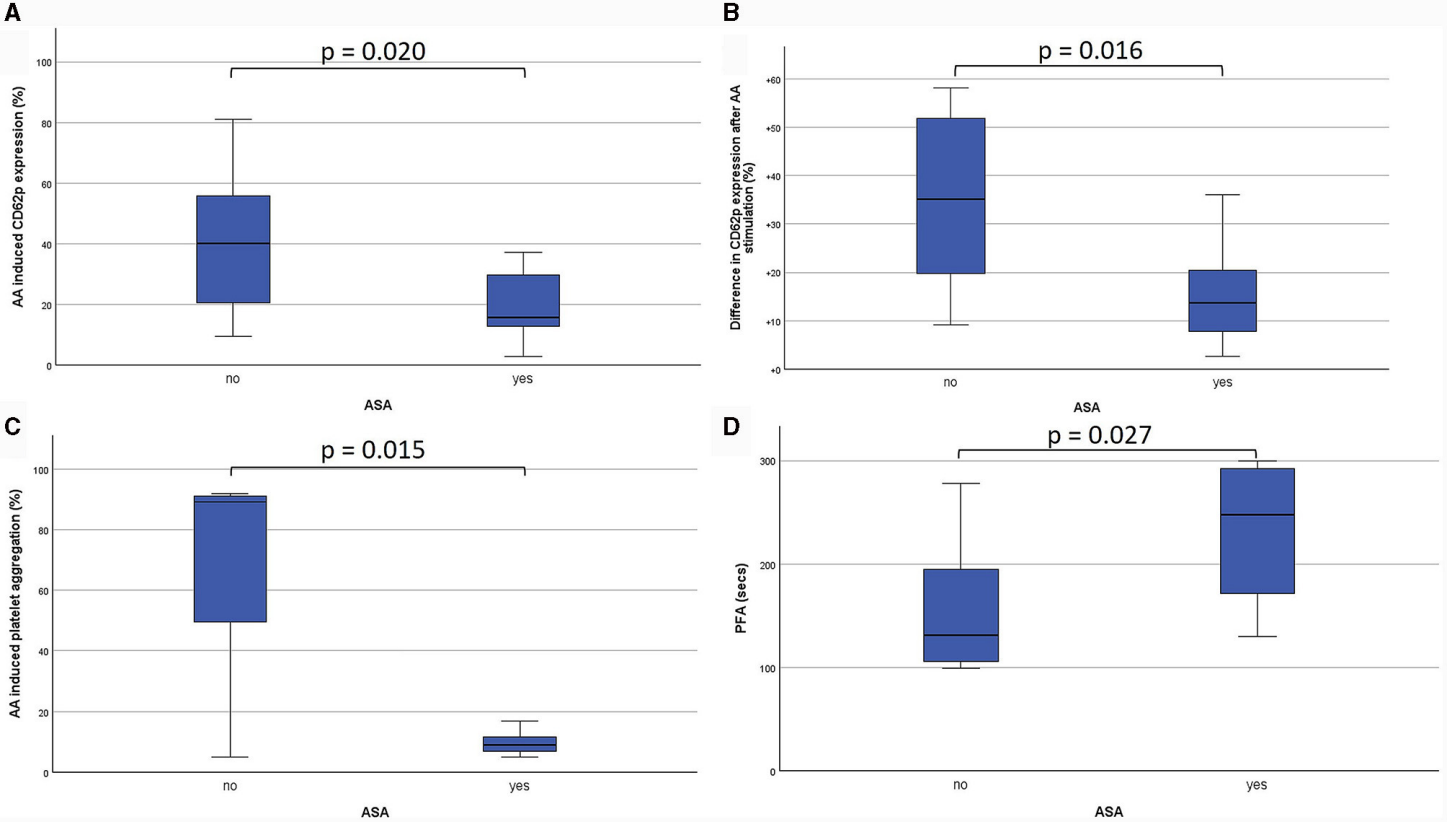
Comparison of the effects of acetylsalicylic acid (ASA) on different platelet functioning parameters. Firstly, CD62-expression is demonstrated as percentage of all platelets (CD61-positive cells) after stimulation with arachidonic acid (AA; **A**). Additionally, the difference between unstimulated and AA-stimulated CD62p-expression on CD61-positive cells is shown **(B)**. Light transmission aggregometry (LTA) revealed the percentage of platelet aggregation after stimulation with AA **(C)**. Platelet functioning analyzer (PFA-200) measurements show the time to plug formation after stimulation with epinephrine **(D)**.

**Figure 4:**
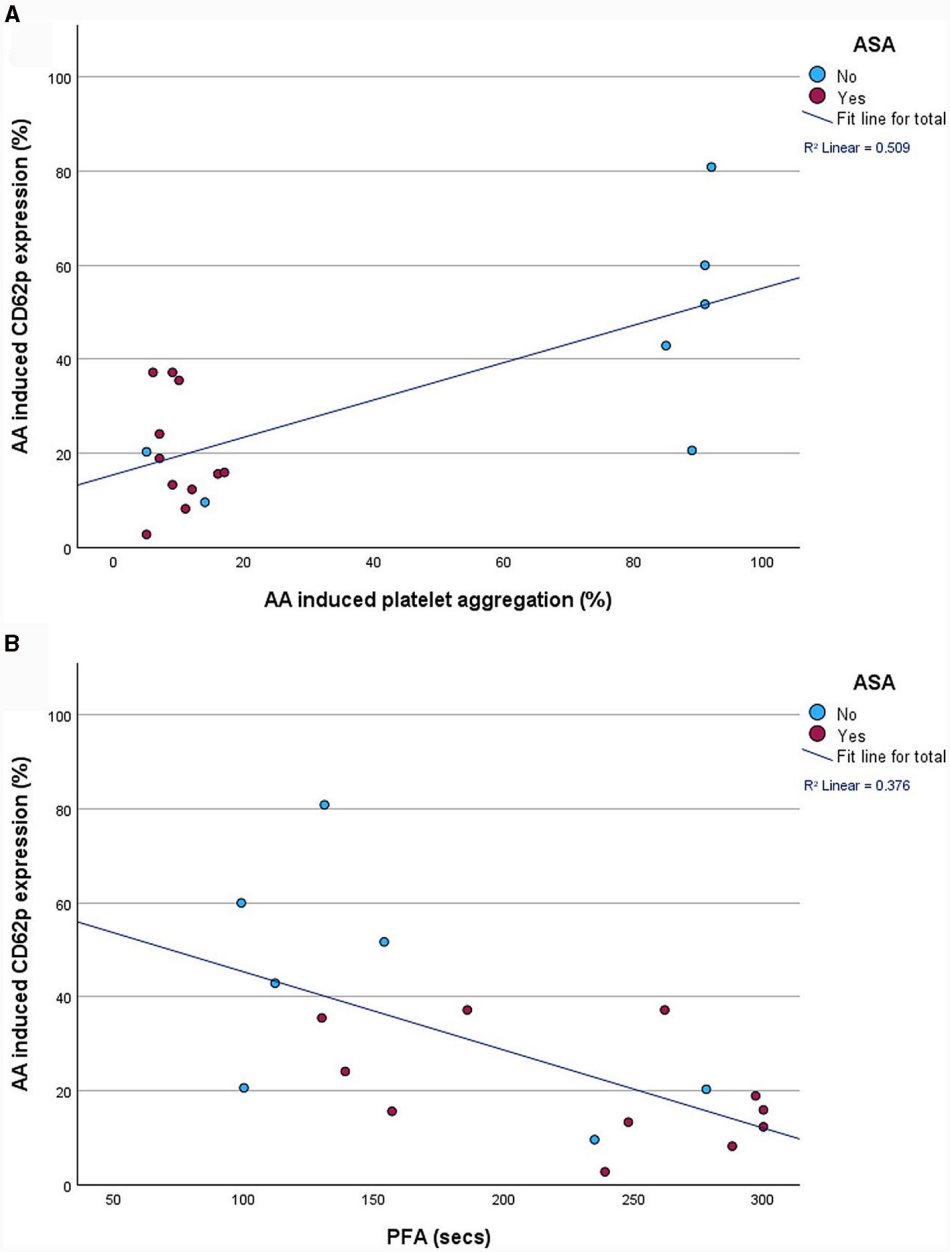
Correlation between fluorescence-activated cell sorting (FACS)-analysis of platelet activation, as measured by percentage of CD62p-positive cells, and aggregometry **(A)** as well as platelet functioning analyzer (PFA-200) plug formation time **(B)**.

PFA-200 and LTA results were obtained in a median time of 149 min (IQR 129.0–146.5 min) after venipuncture. LTA revealed lower platelet aggregation in ASA-pretreated patients compared to no APT (9.0%; IQR 7.0–12.0% vs. 88.5%; IQR 11.8–92.0%; *p* = 0.015). And PFA showed and longer time to plug formation with ASA compared to without (248.0 s; IQR 157.0–297 s vs. 121.5 s; IQR 99.8–174.3 s; *p* = 0.027; Table 2; Figure 3). Significant correlations were noted between AA-induced CD62p expression and aggregometry analysis (*n* = 18; ρ = 0.714; *p* < 0.001 as well as a negative correlation between CD62p increase and PFA clot formation time (*n* = 18; ρ = −0.613; *p* = 0.007; Figure 4). In the ROC-curve analysis, the sensitivity and specificity for ASA intake was highest for PFA (cut-off value ≥ 155.5; sensitivity 81.8%; specificity 71.4%). The respective areas under the curve (AUC) were for FACS-CD62p 0.212 (95%-CI 0.000–0.424; *p* = 0.030), for LTA 0.232 (95%-CI 0.000–0.491; *p* = 0.044) and PFA 0.838 (95%-CI 0.661–1.000, *p* = 0.011).

**Table 2:** Overview of fluorescence-activated cell sorting (FACS), light transmission aggregometry (LTA), and platelet-functioning analyzer (PFA-200) results for patients with and without acetylsalicylic acid (ASA) pretreatment.

		**No ASA**	**ASA**	***P***
FACS (% CD62p-positive cells; IQR)	Unstimulated	2.7% (0.7–6.9%)	2.1% (1.0–4.2%)	0.692
	AA-stimulated	40.1% (12.6–37.2%)	15.8% (20.3–56.3%)	0.020
LTA (%; IQR)		88.5% (11.8–2.0%)	9.0% (7.0–12.0%)	0.015
PFA-200 (s; IQR)		131 s (100–235 s)	248 s (157.0–297.0 s)	0.027

For FACS, the median percentage of CD61-positive cells (platelets), which expressed CD62p without or with stimulation with arachidonic acid (AA) is shown. For LTA, platelets activation is shown as percentage of aggregation after stimulation with AA. For PFA-200, the time to plug formation was measured in seconds (s). Significant group differences for ASA-intake were observed with all three methods (Mann–Whitney–*U*-test).

## Discussion

The present study outlines the feasibility of three different methods to study platelet activation in acute stroke patients to evaluate the effects of preexisting antiplatelet therapy. The *in vitro* measurement of platelets presents a particular challenge due to the rapid degranulation of thrombocytes after venipuncture as well as several other confounding mechanisms such as prolonged blood stasis and transport artifacts (23). Hence, utmost care was taken in our study to ensure minimal platelet pre-activation and rapid transport times. Expected values for CD62p-expression in patients with ischemic stroke within 24 h in previous studies were reported between 1 and 5%, which is comparable to our observation (median 2.21%; IQR 0.98–5.2%) (24, 25). The significant correlation between FACS results and aggregometry as well as PFA highlights the clinical utility of the chosen methods. Some outliers with unexpected low platelet reactivity despite no APT or high reactivity despite ASA intake were observed, which point toward secondary effects on platelet reactivity such as preceding chemotherapy or non-compliance, respectively.

From our results it becomes evident why aggregometry is oftentimes considered the gold standard for the measurement of platelet function as it demonstrated the highest significance in detecting differences between patients with aspirin and without antiplatelet treatment. However, in the exploratory ROC-curve analysis, PFA ultimately demonstrated the highest sensitivity to predict ASA pretreatment. Due to the small sample size, this finding should be regarded cautiously. The positive correlation between FACS results and the routine diagnostic methods allows for the possibility of future implementation, especially if a standardized approach for *in-vitro* platelet stimulation and FACS protocols can be established.

Another relevant discussion point for future trials would be the necessary waiting time to obtain results from these methods and whether it would be feasible to base therapeutic decisions on specific test results. Whereas, it appears hardly reasonable to wait for thrombocyte functioning tests for intravenous thrombolysis in acute ischemic stroke, therapeutic decisions regarding the use of thrombocyte concentrate or desmopressin before surgical approaches in intracerebral hemorrhage occurring under APT might be a realistic scenario (26). In our study, time to results differed between FACS (168 min; IQR 159.5–206.0 min) and the composite of LTA and PFA (149 min; IQR 129.0–146.5 min). Even more rapid platelet functioning testing can be obtained with thrombelastography, though available devices lack standardization (27, 28).

In previous studies, FACS of specific coated platelets, which exhibit procoagulant proteins and phosphatidylserine, has been investigated as a tool to differentiate between hemorrhagic and thrombotic stroke with notable results, highlighting the potential of this method (29). Interestingly, in a study of patients with acute ischemic stroke a weaker response of CD62p expression on platelets to stimulation with thrombin was observed compared to healthy individuals, which was attributed to cleavage of the protease-activated receptor-1 (30). This might constitute another mechanism by which patients with acute stroke are more prone to hemorrhagic complications, which might be enhanced by APT. Another advantage of FACS is the possibility to adjust for pre-activation of platelets, so regardless of stroke type it is possible to determine the quantity of additional *in-vitro* stimulability. To achieve this, it is essential to use an appropriate dose of stimulant (in this case AA or ADP). In our study, the use of thrombin lead to a maximum stimulation, which did escape pretreatment with ASA, hence AA and ADP should be preferred agents for platelet stimulation *in vitro*. In addition to clinical tests, the successful implementation of FACS analyses have been described in experimental animal studies, for which is well-suited due to the limited demand of blood volume required for testing (31).

Strength of our study include the measurement of platelet activation as a percentage of circulating platelets, which was ensured by staining for CD61. Interestingly, antibody staining for PAC1 showed higher pre-activation and markedly less activation in response to AA compared to ADP, suggesting different mechanisms in the recruitment of the glycoprotein (GP) IIb/IIIa complex in response to AA and ADP. Further research could potentially elicit the underlying mechanisms. Meanwhile, if platelet reactivity after ASA intake is the focus of interest, PAC-1 seems to bear little relevance, but is potentially more useful to evaluate the effectiveness of GP IIb/IIIa antagonists such as clopidogrel, prasugrel, or ticagrelor.

## Limitations

During the course of measurements, it became apparent that the laser signal intensity of the channel detecting PE (CD62p) was set too low, resulting in values below 0 in unstimulated samples. However, since platelets still demonstrated significant activity levels, especially compared to isotype control, we continued the measurements with the identical settings to maintain continuity. Another limitation of this study was the single measurement of thrombocyte activation markers after stroke, considering that in a previous study fluctuations were observed in the time period after a stroke index event, most notably showing a rapid decrease in CD62p-expression within 2 weeks (25). As the peak of CD62p-positive platelets was observed on day 1, it seems to be the most appropriate timepoint for measurement, however, it remains unclear how fast changes occur in the immediate period within 24 h after stroke.

To our knowledge, this work represents the first description of multiple platelet activation tests in acute stroke patients. Considering the effort to establish standardized measuring techniques for clinical trials, the comparison of FACS with aggregometry and PFA represents a valuable contribution for future research. From the clinical perspective, it is desirable to establish a reliable, standardized method to determine thrombocyte function in individual patients, which might guide therapeutic decisions in cases with intracranial hemorrhage such as the administration of desmopressin or platelet transfusions (26). The results from this study are likely to guide future projects by providing estimates of the effect size of ASA on different platelet functioning tests as well as highlighting the feasibility and time frame of performing these tests in the setting of acute stroke. By enrolling more patients with different subtypes of stroke, a correlation of clinical outcomes with laboratory parameters can be envisaged. Ideally, these tests would also provide a basis for timely detection of non-responders of APT, which might be at increased for thrombotic complications such as recurrent strokes.

## Conclusion

In the setting of acute stroke, it is feasible to use differentiated platelet analytics to determine alterations caused by antiplatelet therapy. Among the tests under investigation, PFA showed the highest sensitivity for the intake of ASA in stroke patients. FACS analysis, particularly of CD62p-expression after *in-vitro* stimulation with arachidonic acid, on the other hand might be able to provide a more nuanced approach to altered platelet reactivity and prove beneficial for clinical and preclinical research.

## Data availability statement

The raw data supporting the conclusions of this article will be made available by the authors, without undue reservation.

## Ethics statement

The studies involving humans were approved by Ethics Committee of the Goethe University Frankfurt. The studies were conducted in accordance with the local legislation and institutional requirements. The participants provided their written informed consent to participate in this study.

## Author contributions

JS: Conceptualization, Formal analysis, Funding acquisition, Investigation, Methodology, Writing—original draft, Writing—review & editing. FL: Investigation, Methodology, Writing—review & editing. HU: Formal analysis, Investigation, Methodology, Writing—review & editing. FB: Resources, Writing—review & editing. FG: Formal analysis, Resources, Writing—review & editing. WM: Methodology, Resources, Supervision, Writing—review & editing.
